# Effects of fasudil on glial cell activation induced by tooth movement

**DOI:** 10.1186/s40510-024-00518-2

**Published:** 2024-07-22

**Authors:** Wenyuanfeng Chen, Yuan Qu, Yining Liu, Guorui Zhang, Hasan M. Sharhan, Xinzhu Zhang, Kunwu Zhang, Baocheng Cao

**Affiliations:** 1 Department of Orthodontics and Dentofacial Orthopedics, School of Stomatology, Lanzhou University, 222 TianShui South Road, Lanzhou City, 730000 China; 2 International Campus, Zhejiang University-University of Edinburgh Institute, Zhejiang University, Haining, 314400 China

**Keywords:** Orthodontic pain, ROCK inhibitor, Microglia, Astrocyte, Cytokines, Fasudil

## Abstract

**Background:**

Orthodontic pain affects the physical and mental health of patients. The spinal trigeminal subnucleus caudalis (SPVC) contributes to the transmission of pain information and serves as a relay station for integrating orofacial damage information. Recently, glial cells have been found to be crucial for both acute and maintenance phases of pain. It has also been demonstrated that rho kinase (ROCK) inhibitors can manage different pain models by inhibiting glial cell activation. Here, we hypothesized that orthodontic pain is related to glial cells in the SPVC, and Fasudil, a representative rho/rock kinase inhibitor, can relieve orthodontic pain by regulating the function of glial cells and the related inflammatory factors. In this study, we constructed a rat model of tooth movement pain and used immunofluorescence staining to evaluate the activation of microglia and astrocytes. Quantitative real-time PCR was used to detect the release of related cytokines and the expression of pain-related genes in the SPVC. Simultaneously, we investigated the effect of Fasudil on the aforementioned indicators.

**Results:**

In the SPVC, the expression of c-Fos peaked on day 1 along with the expression of OX42 (related to microglial activation), CD16 (a pro-inflammatory factor), and CD206 (an anti-inflammatory factor) on day 3 after tooth movement, followed by a gradual decrease. GFAP-staining showed that the number of activated astrocytes was the highest on day 5 and that cell morphology became complex. After Fasudil treatment, the expression of these proteins showed a downward trend. The mRNA levels of pro-inflammatory factors (IL-1β and TNF-α) peaked on day 3, and the mRNA expression of the anti-inflammatory factor TGF-β was the lowest 3 days after tooth movement. Fasudil inhibited the mRNA expression of pain-related genes encoding CSF-1, t-PA, CTSS, and BDNF.

**Conclusion:**

This study shows that tooth movement can cause the activation of glial cells in SPVC, and ROCK inhibitor Fasudil can inhibit the activation of glial cells and reduce the expression of the related inflammatory factors. This study presents for the first time the potential application of Fasudil in othodontic pain.

**Supplementary Information:**

The online version contains supplementary material available at 10.1186/s40510-024-00518-2.

## Background

Orthodontic discomfort, with an incidence as high as 95%, is a prevalent complication in orthodontic treatment and a major deterrent for patients [1]. The spinal trigeminal subnucleus caudalis (SPVC) is the relay station responsible for integrating nociceptive information from the orofacial nerve [2]. Glial cells have been linked to craniofacial pain [3]; they can cause pain and pain amplification following peripheral activation through chemotaxis, pro-inflammatory cytokine production, and nociceptive signaling via glial–neural interactions [4]. Microglia are considered to be involved in the initiation of pain sensation, whereas astrocytes may be involved in the maintenance and amplification of pain [5, 6]. Although activated glial cells have been studied in spinal neuropathic pain models [7], literature on orthodontic pain models is limited, and whether glial cells in the SPVC are involved in orthodontic pain and its related mechanisms remains unclear.

Non-steroidal anti-inflammatory drugs (NSAIDs) are used as over-the-counter drugs of choice for orthodontic pain [8]. However, they affect bone remodeling and reduce tooth movement. Additionally, although NSAIDs relieve pain, their long-term use may result in chronic pain [9], thereby making their use in orthodontic pain management controversial. Therefore, alternative medications are necessary.

Rho kinase (ROCK) is crucial for the development of inflammation-induced neuropathic pain [10]. Kazuya reported that the activation of ROCK in spinal cord microglia is crucial to initialize neuropathic pain [11]. Keiichiro provided evidence that pro-inflammatory TNF-α promotes CSF-1 production in a ROCK-dependent manner [12, 13] and the release of CSF-1 can induce glial cell proliferation and abnormal mechanical pain in animals [14, 15]. The upregulation of the sensory neuron-CSF-1-glia-BDNF axis and other pain-related genes have been validated in various pain models [16, 17]. Thus, RhoA/ROCK is crucial for inflammatory pain development. In addition, studies have shown that RhoA/ROCK is associated with the morphology of glial cells [18–20].

Fasudil, a representative ROCK inhibitor, has been evaluated in neuropathic pain, inflammatory pain, and mechanical pain models and has been shown to inhibit the activation of microglia and pro-inflammatory factors and upregulate the expression of anti-inflammation-related factors [21, 22]. Based on the results of hot-plate and abdominal constriction response (writhing) tests, Büyükafşar et al. [23] reported that the Rho-kinase inhibitor Y-27,632 represents a new type of antinociceptive drug. Wang et al. [24] demonstrated that by inhibiting the Rho/Rho kinase pathway using Fasudil, LPS-induced hyperalgesia and release of TNF-α and IL-1β in the mouse spinal cord could be prevented.

To our knowledge, the application of ROCK inhibitors in orthodontic pain models has not been reported. Based on the findings of previous research, we aimed to explore the effects of the ROCK inhibitor Fasudil on glial cell activation, inflammatory factors, and pain-related gene expression in a rat model of orthodontic pain.

## Methods

### Animals

All experimental procedures involving animals were approved by. This study adhered to the updated ARRIVE 2.0 guidelines for conducting animal research. Seventy-five adult male Sprague–Dawley (SD) rats (weighing approximately 200–250 g) were purchased from the. The rats were raised in a specific pathogen-free animal laboratory under controlled temperature and humidity with a 12-/12-h light–dark cycle. Adequate water and food were provided to each rat. The rats were randomly allocated to the sham (sham, *n* = 25), tooth movement (TM, *n* = 25), and tooth movement with Fasudil (TM + F, *n* = 25) groups. The samples were collected from each group at five time points (*n* = 5): days 1, 3, 5, 7, and 14 after tooth movement.

### Experimental tooth movement

The rats were sedated via an intraperitoneal injection of 10% chloral hydrate (0.002 mL/kg). Tooth movement in the TM and TM + F groups was induced according to a previously described method [25]; particularly, a nickel–titanium (Ni–Ti) tension spring (0.010 mm × 6 mm) was fixed between the left maxillary first molar and the upper incisor (Figure A1). A measured force of 60 *g* was applied on each rat to simulate orthodontic pain. No force was applied on rats in the sham group. The tension spring was checked once a day for detachment and promptly reinstalled, if detached.The distances of tooth movement were measured at 7 and 14 days using a gap-measuring ruler. (Table A1)

### Von frey mechanical pain threshold test

Pain thresholds were measured on days 1, 3, 5, and 7 with Von Frey filaments ranging from 1 g to 16 g, applied on the tooth movement side of the face, aiming for the region including the vibrissae to the point in front of the eyes. Positive responses included asymmetric scratching, attempts to bite the filament, and rapid head movements to avoid the stimulus. A positive result was noted if any of these behaviors occurred. The test was conducted three times with a 10-second interval. The minimum force that elicited two out of three positive responses was recorded as the pain sensitivity threshold. (Figure A2)

### Fasudil and saline treatments

The rats in the TM + F group (*n* = 25) were intraperitoneally administered 30 mg/kg Fasudil hydrochloride dissolved in 0.9% sterile saline (Aladdin, Shanghai, China) daily during tooth movement. The dose and technique of Fasudil administration used were as previously described, and they have been proven to have no effect on rat health [26]. The rats in the sham group were injected the same dose of saline daily.

### Immunofluorescence staining

On days 1, 3, 5, 7, and 14 after tooth movement, the rats were anesthetized via intraperitoneal administration of an excessive dose of 10% chloral hydrate until respiration was substantially inhibited. Thereafter, their blood vessels were perfused with 200 mL of 0.1 M phosphate-buffered saline and then with 4% paraformaldehyde, followed by immediate removal of the medulla oblongata, which was placed in 4% paraformaldehyde solution overnight at 4 °C. The medulla oblongata was cut into coronal slices of 30-µm thickness using a vibrating microtome, and we followed “The Rat Brain in Stereotaxic Coordinates − 7th Edition” to find the SPVC region (Figure A3). All measurements and observations of immunofluorescence staining were performed in this region. The brain slices were rinsed thrice with phosphate-buffered saline (PBS) for 5 min; the membrane was ruptured using 0.5% PBS-Triton X 100 for 10 min, and then blocked with goat serum at 37 °C for 1 h. The brain slices were rinsed with PBS for 10 min. The slices were incubated overnight at 4 °C with the following primary antibodies: anti-c-Fos (c-Fos: Abcam; 1:1600, No: ab208942), anti-OX42 (OX42: Abcam; 1:500, No: ab1211), anti-GFAP (GFAP: Cell Signaling Technology; 1:500, No: 3670), anti-CD16 (CD16: Abcam; 1:500, ab211151), and anti-CD206 (CD206: Cell Signaling Technology; 1:500, No: 24,595). After incubation, the fluorescent-labeled secondary antibodies rhodamine (TRITC)-conjugated Goat Anti-Rabbit IgG (H + L) (Proteintech; No: SA00007-2) and fluorescein (FITC)-conjugated AffiniPure Goat Anti-Mouse IgG (H + L) (Proteintech; No: SA00003-1) were used. The nuclei were stained with Hoechst 33,258 (1:10,000; Sigma) for 10 min.

We evaluated the immunoreactivity of OX42, GFAP, and c-Fos quantitatively. All brain slices were first observed under a fluorescent microscope (BX51; Olympus, Tokyo, Japan) before being photographed; the photographs were saved in the TIFF format using a charge-coupled device camera (XZ-1; Olympus). Using Image J software, we binarized the images and carried out automatic counting of positive cells, a technique that has been demonstrated to have important benefits in terms of consistency, accuracy, and dependability [27]. We used the mean of the outcomes following three blinded measurements.

### Quantitative real-time polymerase chain reaction (PCR)

The rats were sacrificed via intraperitoneal injection of a lethal dose of chloral hydrate, and their medulla oblongata was immediately removed, frozen in liquid nitrogen, and stored at − 80 °C. The total RNA was extracted using the TRIzol reagent (Takara; code no: 9108) and reverse-transcribed to cDNA using the PrimeScript RT kit (Takara; code no: RR036A). The PCR primers were designed and synthesized by Goldwisdom Biotechnology Co, Ltd. Quantitative PCR was performed using the SYBR Green Supermix (Takara; code no: RR820B). The 2-ΔΔCt method was used to analyze the mRNA expression of CSF-1, BDNF, CTSS, t-PA, TNF-α, IL-1β, and transforming growth factor-β (TGF-β) in the SPVC. The reference genes used for the analysis are displayed in Table 1.

**Table 1:** Sequence of primers used in the polymerase chain reaction

Gene	Forward (5´–3´)	Reverse (5´–3´)
*TNF-α* *IL-1β* *TGF-β*	ATGGCCTCCCTCTCAGTTC CTTCAAATCTCACAGCAGCA AGGCGGTGCTCGCTTTGTA	TTGGTGGTTTGCTACGACGTG AGCAGGTCGTCATCATCCCAC TCCCGAATGTCTGACGTATTG
*CSF-1*	CTGCCCTTCTTCGACATG	GTAGCAAACGGGATCGTC
*CTSS*	CTGCCCTTCTTCGACATG	AGTGAAGGCCCCAACTGT
*t-PA*	GCCCTGACGGATTTGTTG	CCCAGCTTGATGGCATTT
*GAPDH*	TGAACGGGAAGCTCACTGG	TCCACCACCCTGTTGCTGTA

### Sholl morphological analysis of astrocytes

For the specific detection of morphological changes in astrocytes, we selected 60 astrocytes (four cells from each rat of the three groups) [28]. Figure 4D illustrates the Sholl analysis findings of astrocytes, which were labeled with GFAP antibody. The three-dimensional (3D) modeling of GFAP-stained astrocytes was performed using ImageJ software-volume viewer (Java; NIH, USA) [29], and then binary masks representing astrocyte processes were created for Sholl analysis. Sholl analysis was performed by overlaying circles of increasing diameter (in 5-µm steps as reported previously [28]), from the center of the soma of an astrocyte and by counting the intersections that the astrocyte makes with each circle.

### Statistical analysis

Data analysis was performed using GraphPad Prism version 9.0. All statistical analyses were performed under double-blind conditions, and all data were subjected to a one-way ANOVA and two-tailed *t-*test. Data are expressed as mean ± standard error. Results with *p* < 0.05 and *p* < 0.01 were considered to be significant and extremely significant, respectively. PASS 2021 software (NCSS, Kaysville, Utah, USA)was used to calculate sample size. A one-way ANOVA with a sample of 75 subjects divided among three groups achieves a power of 81%.

## Results

### Effect of Fasudil on c-Fos expression

**Fig. 1:**
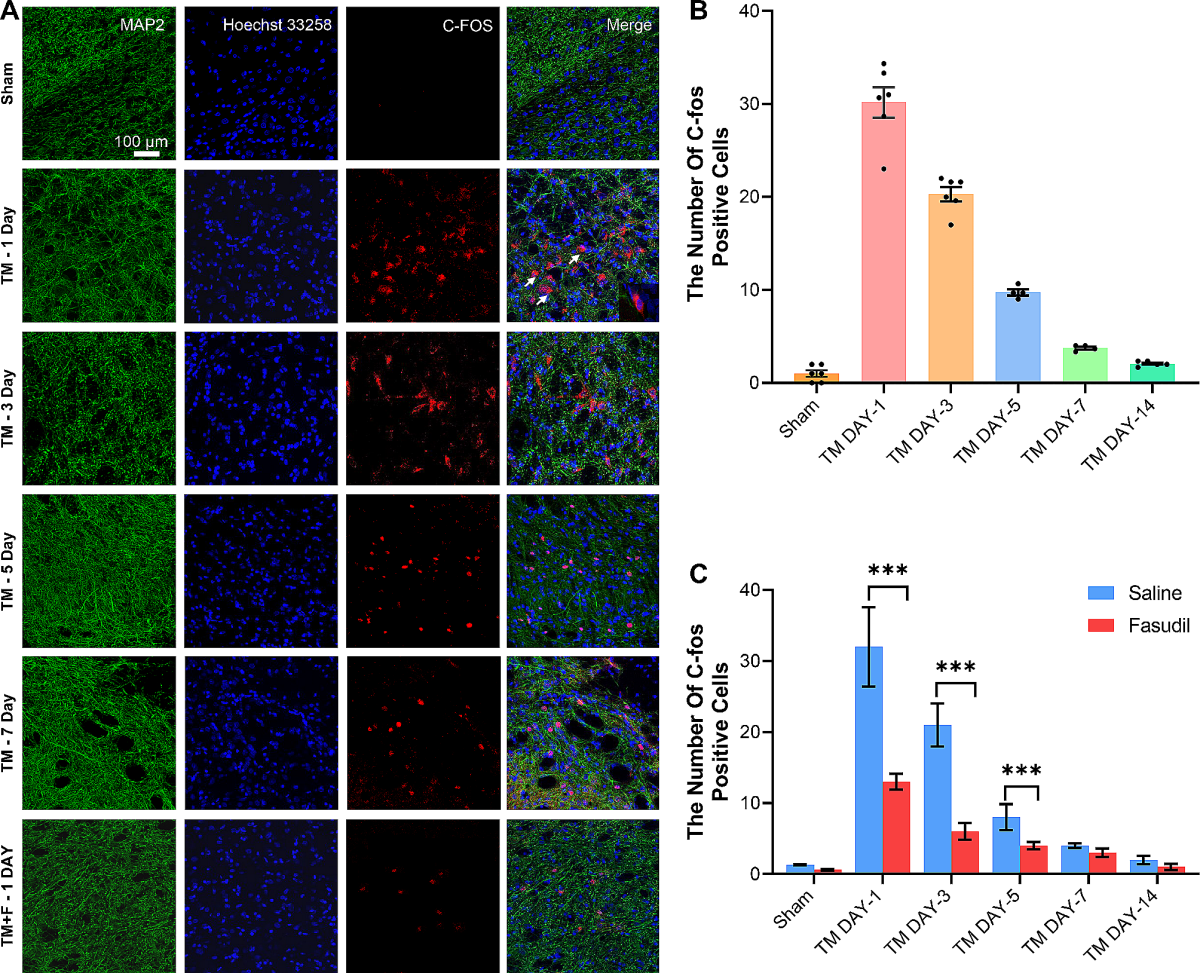
Fluorescence images of c-Fos-immunoreactive neurons in the ipsilateral side of the SPVC. (**A**) c-Fos expression in the sham and TM groups on days 1, 3, 5, and 7 and in the TM ± F group on day 1. (**B**) Statistics of the number of c-Fos-positive cells in the SPVC in the TM group. (**C**) Effect of Fasudil on c-Fos expression in the SPVC compared with that in the TM group. All data are presented as mean ± standard error (*n* = 5). *p < 0.5, **p *<* 0.01, ***p *<* 0.001

We observed the effect of tooth movement on c-Fos (a nociceptive neuron response protein) expression in the SPVC of rats using immunofluorescence staining (Fig. 1); very few c-Fos-immunoreactive cells were observed in the sham group. In the TM group, c-Fos expression peaked on day 1, gradually declined on day 3, and further decreased after day 5. The TM + F group showed significantly fewer c-Fos-positive cells induced by tooth movement on day 1 (*p* < 0.001) after Fasudil administration. These results indicate that c-Fos was significantly activated on day 1 after tooth movement treatment and significantly inhibited after Fasudil administration.

### Effect of Fasudil on tooth movement-induced activation of microglia

**Fig. 2:**
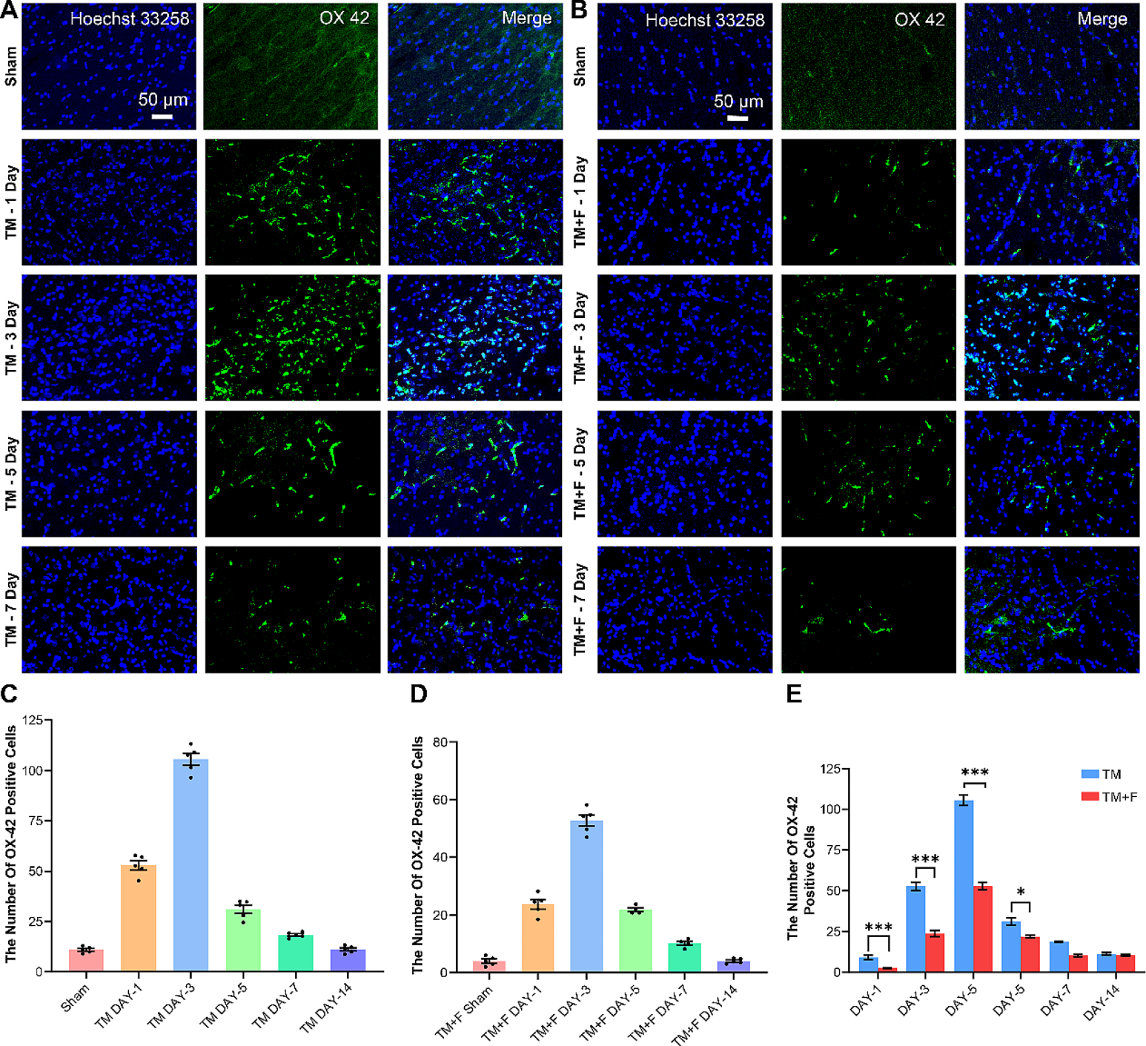
Fluorescence images of OX42 immunoreactivity in the ipsilateral side of SPVC. (**A**-**B**) The expression of OX42 in the sham and TM / TM  ± F groups on days 1, 3, 5, and 7. (**C**-**D**) Statistics of the number of OX42-positive cells in the SPVC of the TM / TM ± F groups on days 1, 3, 5, and 7. (**E**) Effect of Fasudil on OX42 expression in the SPVC compared with that in the TM group. All data are presented as mean ± standard error (*n* = 5). *p < 0.5, **p < 0.01, ***p < 0.001

OX42 is a microglia-specific marker, and we observed the effect of tooth movement on microglia in the rat SPVC. Compared with that in the sham group, obvious OX42 expression was noted on day 1 in the TM group, which peaked after day 3 (Fig. 2). Simultaneously, we observed that the morphology of microglia changed from small and branched in the resting state to large and flat amoeba-like. However, following Fasudil administration, OX42 expression was significantly downregulated on day 3 (*p* < 0.001), suggesting that tooth movement activated microglia in the SPVC, which was significantly inhibited by Fasudil.

### Effects of Fasudil on the polarization state of microglia

**Fig. 3:**
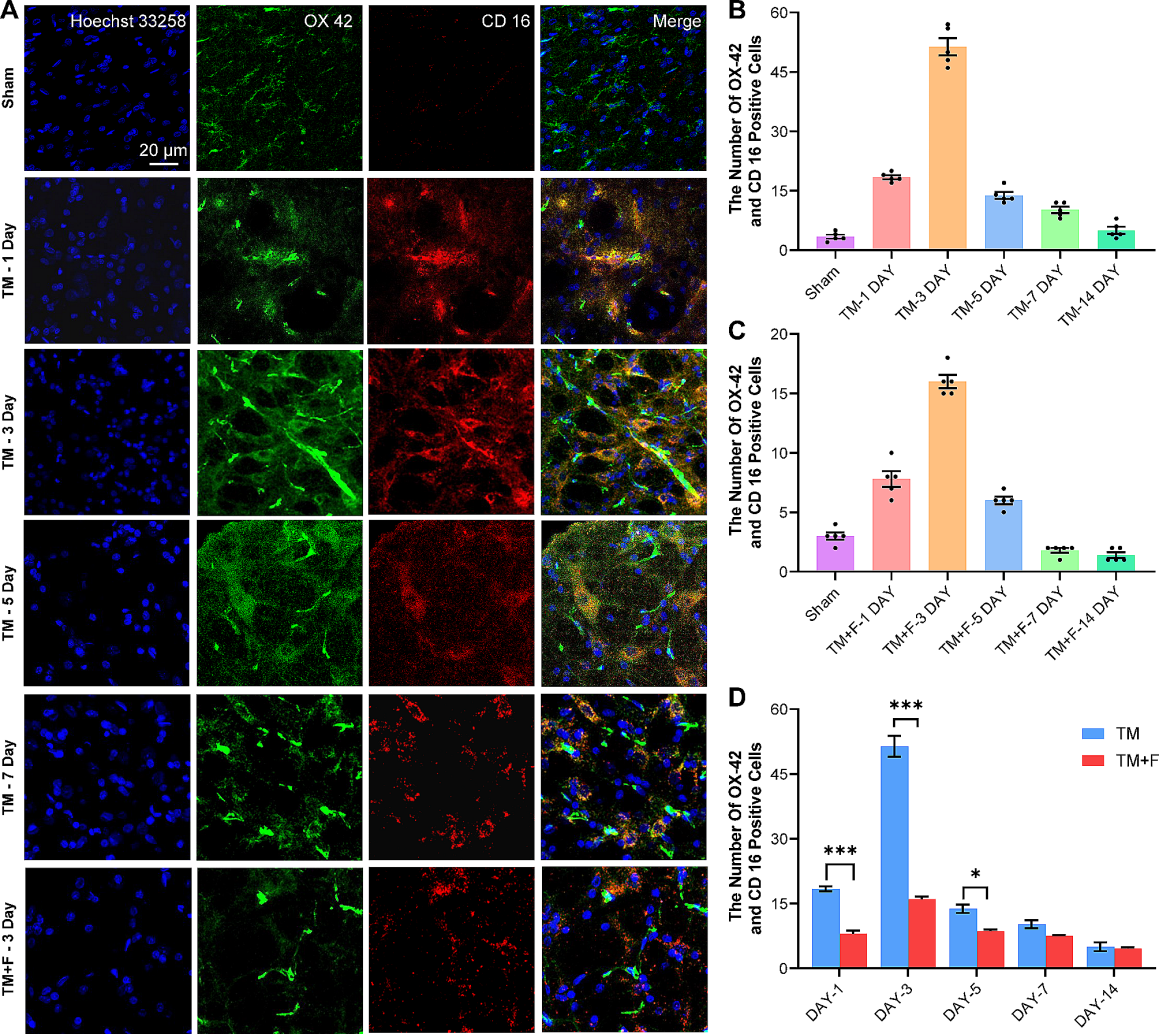
Fluorescence images of CD16 immunoreactivity in the ipsilateral side of the SPVC. (**A**) Expression of CD16 in the sham and TM groups on days 1, 3, 5, and 7 and in the TM + F group on day 3. (**B**) Statistics of the number of CD16-positive cells in the SPVC of the TM group. (**C**) Statistics of the number of CD16-positive cells in the SPVC of the TM + F group. (**D**) Effect of Fasudil on CD16 expression in the SPVC compared with that in the TM group. All data are presented as mean ± standard error (*n* = 5). *p < 0.5, **p < 0.01, ***p < 0.001

**Fig. 4:**
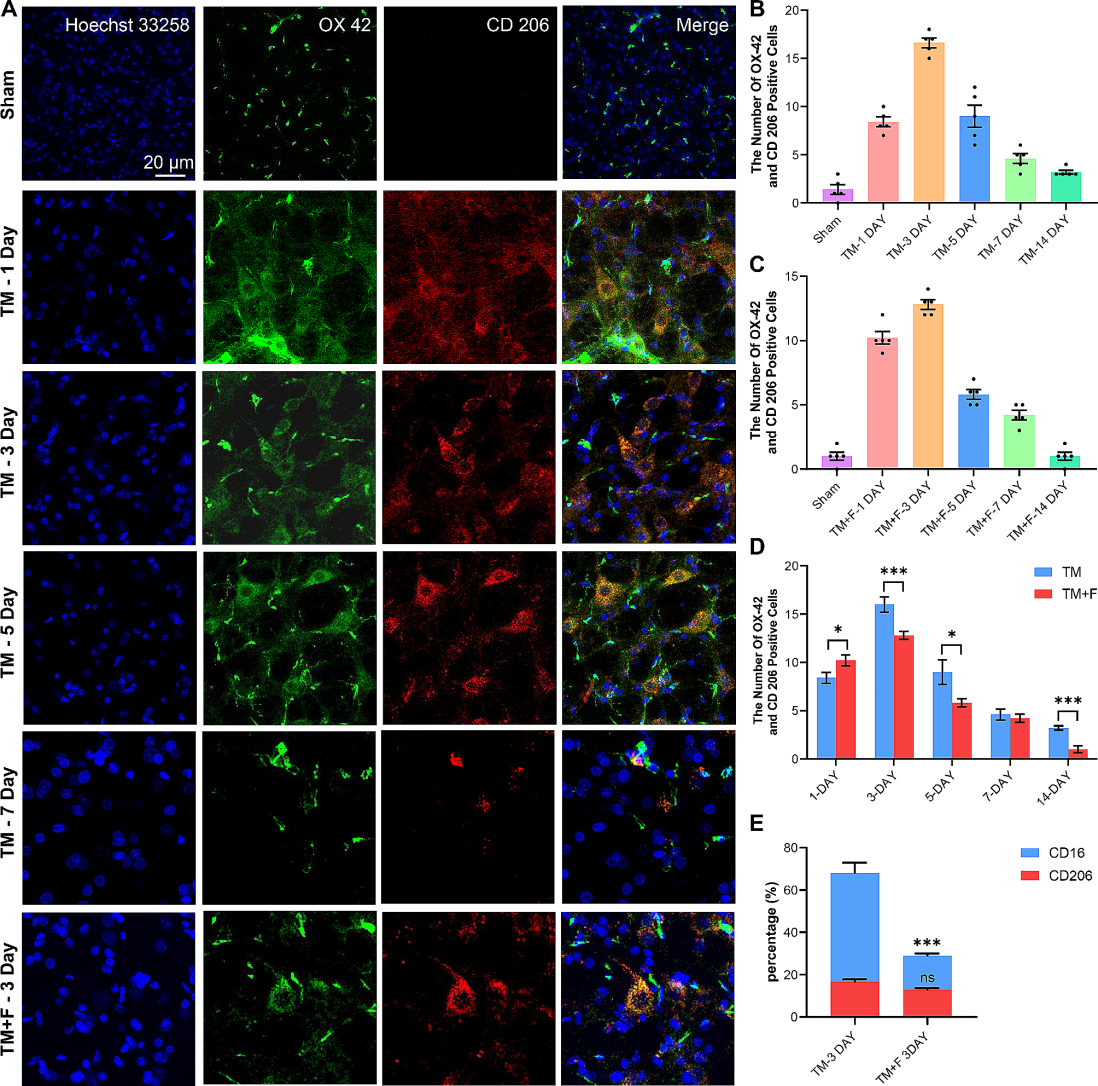
Fluorescence images of CD206 immunoreactivity in the ipsilateral side of the SPVC. (**A**) Expression of CD206 in the sham and TM groups on days 1, 3, 5, and 7 and in the TM + F group on day 3. (**B**) Statistics of the number of CD206-positive cells in the SPVC of the TM group. (**C**) Statistics of the number of CD206-positive cells in the SPVC of the TM + F group. (**D**) Effect of Fasudil on CD206 expression in the SPVC compared with that in the TM group. (**E**) Proportional changes between CD16 and CD206 before and after the administration of Fasudil. All data are presented as mean ± standard error (*n* = 5). *p < 0.5, **p < 0.01, ***p < 0.001

Microglia include the pro-inflammatory M1-type and anti-inflammatory M2-type, which release pro-inflammatory and anti-inflammatory cytokines, respectively. We selected CD16 as the M1-type marker and CD206 as the M2-type marker and observed changes in the polarization state of microglia in the SPVC during tooth movement. In the microglia activated by tooth movement, the main phenotype was the M1-type marker CD16, whereas CD206 was slightly expressed (Figs. 3 and 4). The M1/M2 ratio was altered in the TM + F group compared with that in the TM group, which characterized the anti-inflammatory M2 microglia, and the proportion of CD206 was elevated (*p* < 0.01). This indicates that tooth movement induced M1-dominant microglial activation in the SPVC, whereas Fasudil relatively upregulated the anti-inflammatory M2-type microglia and inhibited microglial activation.

### Effect of Fasudil on tooth movement-induced activation and morphology of astrocytes

**Fig. 5:**
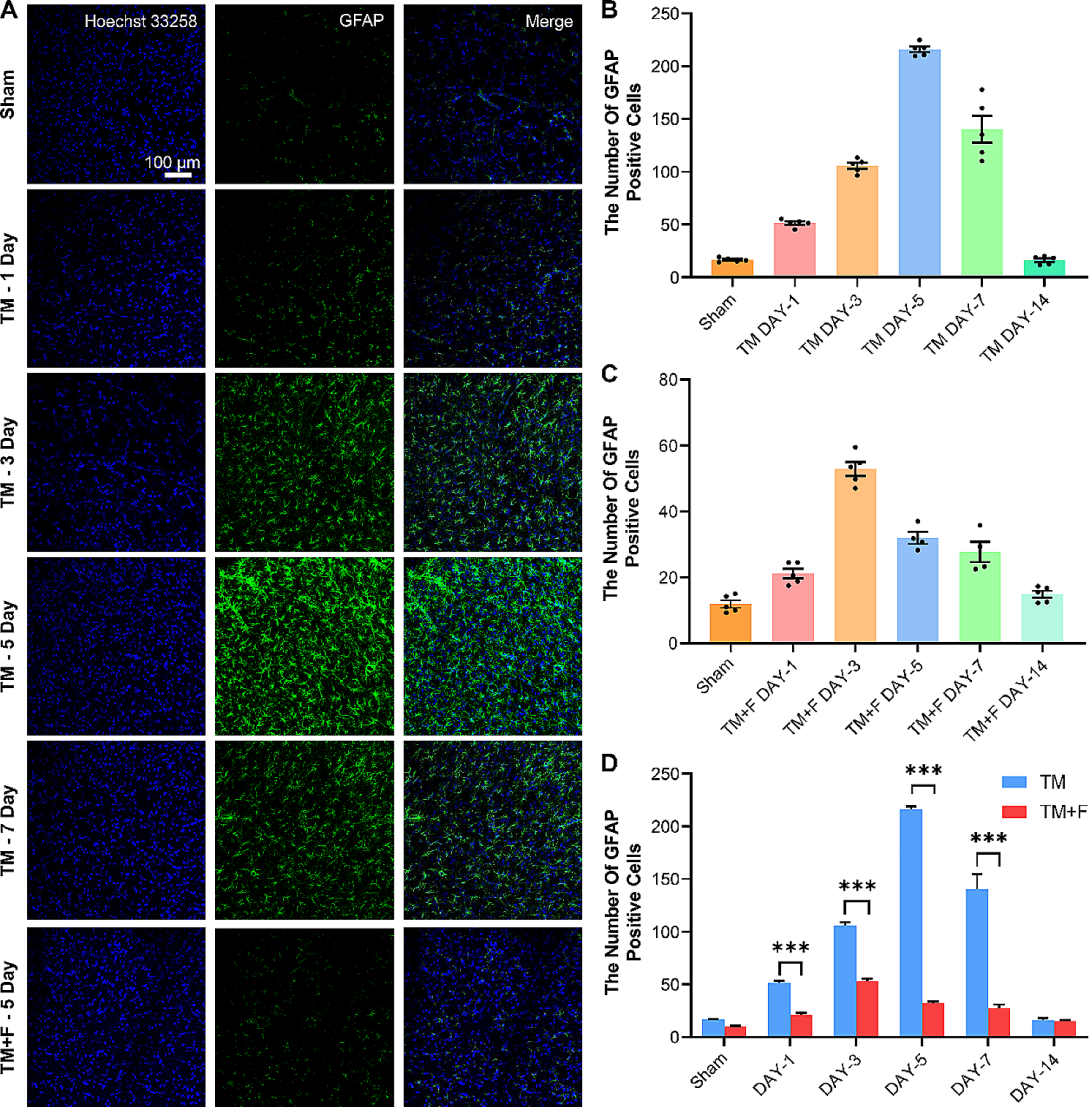
Fluorescence images of GFAP immunoreactivity in the ipsilateral side of SPVC. (**A**) The expression of GFAP in the sham and TM groups on days 1, 3, 5, and 7 and in the TM + F group on day 5. (**B**) Statistics of the number of GFAP-positive cells in the SPVC of the TM group. (**C**) Statistics of the number of GFAP-positive cells in the SPVC of the TM + F group. (**D**) The effect of Fasudil on GFAP expression in the SPVC compared with that in the TM group. All data are presented as mean ± standard error (*n* = 5). *p < 0.5, **p < 0.01, ***p < 0.001

**Fig. 6:**
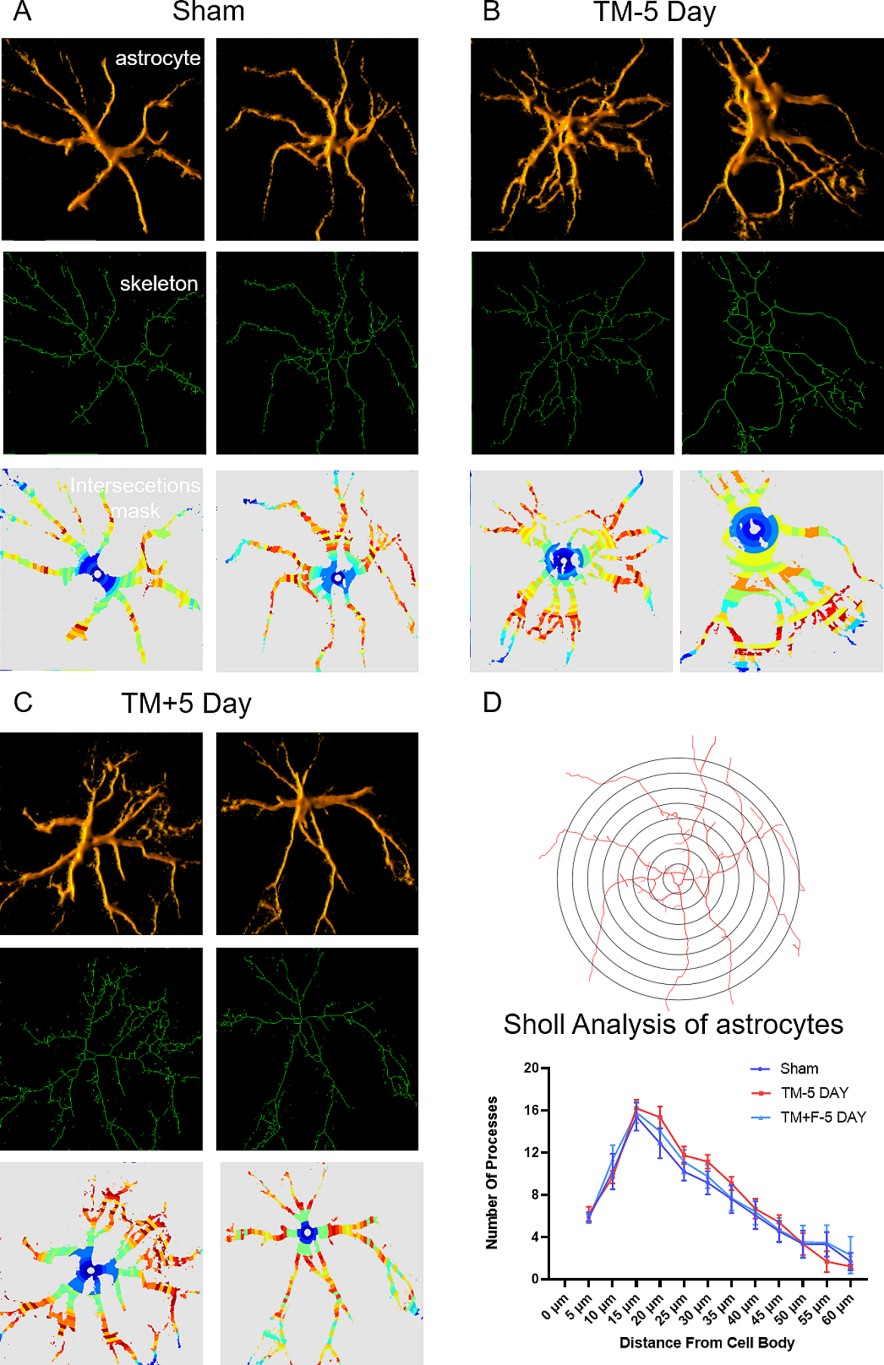
3D modeling of GFAP-stained astrocytes and morphological changes in astrocytes evaluated using the Sholl analysis. (**A**-**C**) Comparison of morphological changes in astrocytes on day 5 between the sham and TM + F groups. (**D**) Quantification of the maximum intersections of the Sholl profile analysis showed the morphological complexity of astrocytes in the sham, TM, and TM + F groups, where the concentric circles had a step distance of 5 μm. All error bars represent mean ± standard error; *n* = 5 animals (4 astrocytes per animal). Scale bar = 20 μm

The role of astrocytes in different pain models has been reported [30]. Here, we characterized astrocytes based on the expression of glial fibrillary acidic protein (GFAP). GFAP expression was upregulated on day 1 of tooth movement and peaked on day 5 (Fig. 5). There was no significant difference in the immunoreactivity of GFAP on day 14, whereas the administration of Fasudil significantly inhibited the expression of GFAP. We further evaluated the morphological changes in astrocytes using Sholl analysis, which uses the nucleus as a dot and records the branches of cells falling on concentric circles of different sizes by drawing concentric circles every 5 μm to assess the complexity of the cell morphology (Fig. 6). On day 5, the number of branches of astrocytes (*p* < 0.01) and cell-body volume increased, and the cell processes had thickened. Following Fasudil administration, GFAP expression was significantly inhibited (*p* < 0.05), whereas the morphological backbone characterized by GFAP returned to a “star shape.” This finding suggests that tooth movement activates and complicates astrocytes in the SPVC, whereas astrocyte activation is inhibited and a quiescent state is restored after Fasudil administration.

### Effect of Fasudil on tooth movement-induced expression of CSF-1, BDNF, CTSS, t-PA, TNF-α, IL-1β, and TGF-β

Using qRT-PCR, we examined the expression of pain-related genes encoding CSF-1, BDNF, CTSS, and t-PA. The CSF-1-microglia-BDNF axis has been validated in different pain models, and CTSS and t-PA may be associated with changes in glial cell morphology. The PCR analysis showed that CSF-1 expression increased on day 1, decreased on day 3, and then increased again on day 5. After Fasudil administration, CSF-1 expression was significantly downregulated on day 1 (*p* < 0.01). BDNF expression peaked on day 1, and then decreased slowly in the TM group. Compared with that in the sham group, BDNF expression was inhibited by Fasudil (*p* < 0.001). CTSS expression peaked on day 3 (*p* < 0.01), whereas high t-PA expression was observed on days 1 and 7 (*p* < 0.001); however, the expression of both was inhibited by Fasudil administration.

**Fig. 7:**
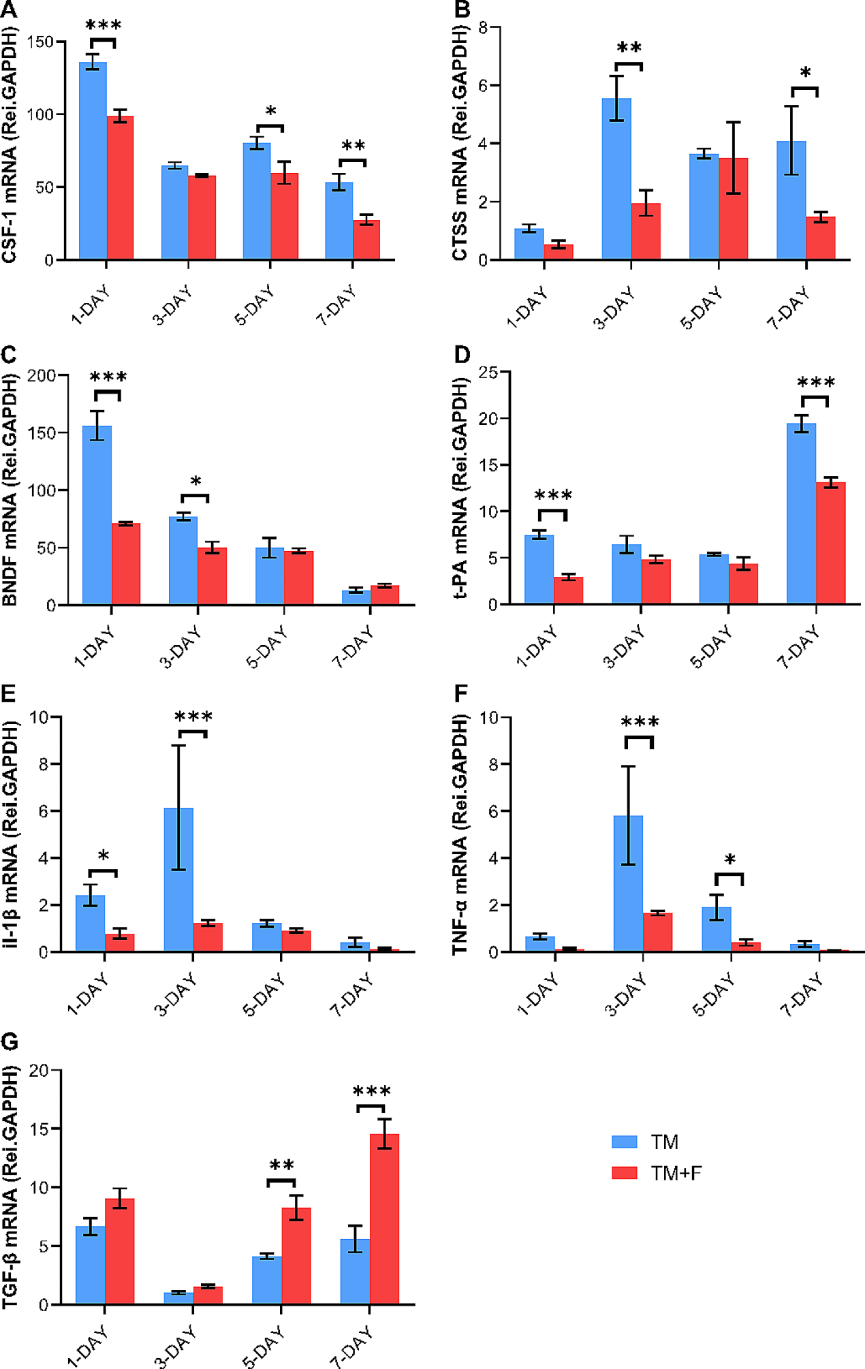
(**A**-**D**) Effect of Fasudil on the expression of pain-related genes encoding BDNF, CSF-1, CTSS, and t-PA in the SPVC compared with that in the TM group on days 1, 3, 5, and 7, determined using real-time polymerase chain reaction. (**E**-**G**) The effect of Fasudil on the expression of the inflammatory factors TNF-α, IL-1β, and TGF-β in the SPVC compared with that in the TM group on days 1, 3, 5, and 7, determined using real-time polymerase chain reaction. All data are presented as mean ± standard error (*n* = 5). *p < 0.5, **p < 0.01, ***p < 0.001

Furthermore, we detected the mRNA expression of inflammatory factors (TNF-α, IL-1β, and TGF-β) during tooth movement. The expression of TNF-α increased on day 3; Fasudil administration inhibited the expression of TNF-α and IL-1β (*p* < 0.001; Fig. 7), whereas the expression of the anti-inflammatory factor TGF-β decreased initially, then increased from days 1 to 3, and after Fasudil administration, it was upregulated on days 5 and 7 (p *<* 0.001). This finding further indicates that Fasudil can promote the expression of anti-inflammatory factors and inhibit pro-inflammatory factors.

## Discussion

**Fig. 8:**
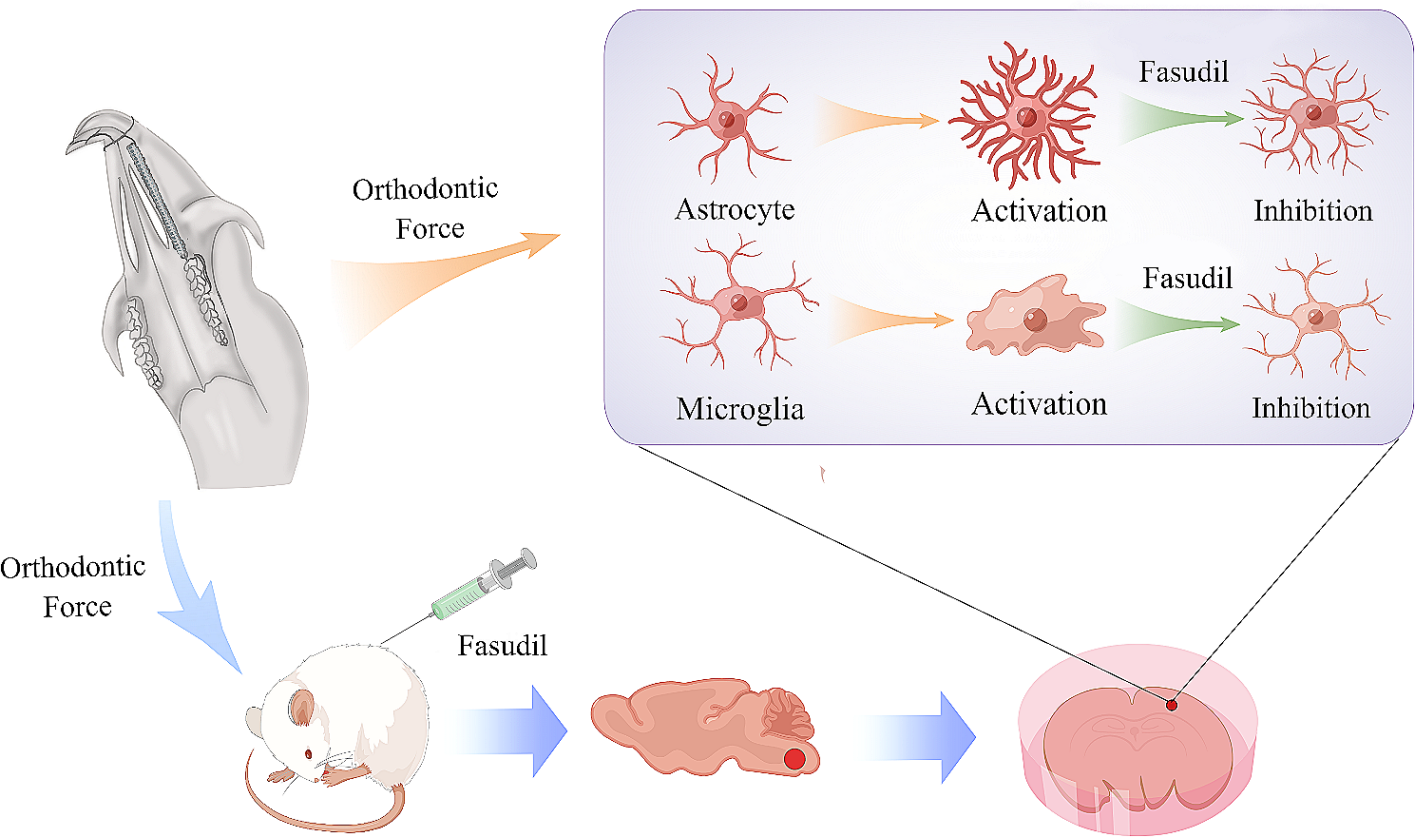
Schematic illustration of orthodontic force mediating the activation of microglia and astrocytes in the SPVC. Microglia were activated into short and thick branches into amoeba-like structures, whereas astrocytes changed from having slender branches to thick branches, with the increase in cell protrusions. After the intraperitoneal injection of Fasudil, glial cells returned to their resting state

The mechanism underlying orthodontic pain remains unclear. Here, we found that c-Fos, microglia, astrocyte were activated/upregulated after tooth movement treatment and inhibited by Fasudil. Fasudil promoted the expression of anti-inflammatory factors and inhibited the expression of pro-inflammatory factors. This study suggests a potential link between orthodontic pain and activation of microglia and astrocytes in the SPVC (Fig. 8).

c-Fos expression peaked on day 1 and then gradually decreased. This finding was consistent with the occurrence and regularity of peak pain in patients receiving orthodontic treatments. Based on a behavioral experiment of orthodontic pain, Sood et al. [31] reported that in the postoperative period of tooth movement (days 1–5), pain peaked on day 1 and then returned to the preoperative level.

We examined CSF-1 and BDNF expressions in the SPVC, noting that CSF-1 expression rose significantly on day 1, decreased on day 3, and increased again on day 5. This pattern aligns with the role of CSF-1 in promoting microglial growth and central sensitization, particularly following peripheral nerve lesions13,17. Fasudil significantly reduced CSF-1 expression, which is consistent with the results of a previous study12. Astrocyte activation may be directly related to the re-upregulation of CSF-1 expression on day 5 [32]. This may be a reason for the transformation of chronic orthodontic pain and central sensitization. As a neurotransmitter and neuromodulator, BDNF activates microglia, resulting in peripheral nerve injury-induced pain. Activated microglia further induce increased BDNF production and upregulate TNF-α release [33]. We found that BDNF expression peaked on day 1, and then decreased slowly, which may be closely related to microglial activation and therefore pain induction.

When orthodontic force is applied, both C and Aδ fibers in periodontal tissue are sensitized [34]. C fiber stimulation activates spinal cord microglia, while Aδ fibers maintain this activation [35]. This study tracks microglial activation by monitoring active microglia numbers during tooth movement. OX42 expression, indicating microglial activation, was prominent on day 3 and gradually diminished over the following 5–7 days.

Concurrently, we noticed morphological alterations of the microglia after just 1 day of activation, demonstrating thick branches and larger cell bodies. OX42 cells displayed a round shape and typical “amoeba-like” morphology on day 3 after activation, and this morphological change may be closely related to CTSS expression in microglia. A significant increase in the CTSS level after the transformation of LPS-induced microglia to amoeba-like cells has been reported [36]. In this study, we found that CTSS expression significantly increased on day 3, consistent with the morphological changes in microglia.

Drugs that block microglial activation, including minocycline, have been developed to treat pain [37]; however, blocking all physiological roles is not ideal. Depending on the physiological milieu, some cytokines and chemicals can polarize activated microglia into either a pro-reparative (anti-inflammatory) M2 state or cytotoxic (pro-inflammatory) M1 state [38]. Therefore, transforming M1-type microglia into M2-type cells may be a sensitive and accurate targeted therapeutic technique. Here, we found both M1-type microglia and M2-positive cells, wherein Fasudil administration caused a decrease in the M1-phenotype cells and an increase in the M2-phenotype cells.

We characterized astrocytes using GFAP and observed differences in their activation timing compared to microglia in orthodontic-induced pain. GFAP peaked on day 5 and gradually declined over the next 7–14 days, which was consistent with the findings of Deguchi [37]. Unlike the continuous activation of microglia (OX42), GFAP upregulation occurred mainly in the chronic pain phase, suggesting a role in persistent pain.

Using GFAP-specific antibodies, we examined astrocyte morphological changes induced by tooth movement, revealing increased branching and complex morphology. This phenomenon has been reported previously [29], and some researchers have used ROCK inhibitors to restore the quiescent state of activated astrocytes [19], Here, Fasudil significantly inhibited GFAP upregulation and restored the star-shaped morphology. On day 7, we observed t-PA upregulation, which is critical for these morphological changes [19]. ROCK inhibitors have been shown to prevent t-PA-induced cytoskeletal changes in astrocytes, consistent with our findings.

We measured TNF-α, IL-1β, and TGF-β expressions, observing a significant rise in IL-1β and TNF-α mRNA levels on day 1, with Fasudil inhibiting these pro-inflammatory factors. This aligns with previous findings showing Fasudil’s downregulation of TNF-α and IL-β in LPS-induced pain [39], which indicate that IL-1β from microglia stimulates astrocytes to produce TNF-α and IL-1β, thereby amplifying the initial IL-1β signal, which may help maintain long-term pain [40]. TGF-β, an anti-inflammatory factor, inhibits IL-1β and TNF-α expression and the proliferation of microglia [41, 42]. Fasudil’s upregulation of TGF-β suggests its role in restoring the balance between anti-inflammatory and pro-inflammatory factors, potentially through inhibiting the NF-κB pathway [41].

It is necessary to choose an appropriate medication to reduce pain caused by orthodontic treatment. ROCK inhibitors plays an important role in various types of pain11. Fasudil has shown positive clinical outcomes and its potential analgesic effects. This makes it a promising candidate for bridging the findings from rat models to potential human applications. The successful translation of these findings may have significant implications for improving pain management and personalized treatment approaches in orthodontics. Further research, including clinical trials, is necessary to validate the effectiveness, safety, and optimal dosage and administration methods of Fasudil in human subjects.

However, orthodontic pain has complex regulatory mechanisms involving many osteoimmunomodulation pathways, the phenomena occurring in the SPVC may also be related to osteoimmunomodulation induced by tooth movement. How to control such variables in future tooth movement pain models remains to be explored. From the perspective of pain, the recruitment of a large number of immune cells by bone immunomodulation releasing inflammatory factors also contributes to pain regulation. In forthcoming research, it is essential to further investigate the extent to which osteoimmunomodulation resulting from tooth movement contributes to the experience of pain. The effects of Fasudil on tooth movement have not yet been elucidated. It has been reported that Fasudil can promote the activation of osteoclasts, however, in this study, no significant impact of fasudil on tooth movement was observed, and its adverse effects on root resorption are yet to be determined.

## Conclusion

To our knowledge, we report for the first time that the Rho/ROCK inhibitor Fasudil reduces the number of nociceptive neurons, reduces the number of M1-type microglia, inhibits astrocytes during tooth movement-induced glial cell activation in the SPVC of rats, and reverts the shape of astrocytes to that in the resting state. The Rho/ROCK inhibitor Fasudil inhibited the expression of pain-related genes encoding CSF-1, CTSS, BDNF, and t-PA, and the pro-inflammatory factors TNF-α and IL-1β at the mRNA level during tooth movement. These results suggest new therapeutic strategies and a theoretical basis for the potential application of Fasudil against orthodontic pain.

### Electronic supplementary material

Below is the link to the electronic supplementary material.

Supplementary Material 1

## Data Availability

Not applicable.

## Abbreviations

3D: three-dimensional
GFAP: glial fibrillary acidic protein
Ni–Ti: nickel–titanium
NSAID: non-steroidal anti-inflammatory drug
PBS: phosphate-buffered saline
PCR: polymerase chain reaction
ROCK: rho kinase
SD: Sprague–Dawley
SPVC: spinal trigeminal subnucleus caudalis
TGF-β: transforming growth factor-β
TM: tooth movement
t-PA: tissue-type plasminogen activator
