# Supplementary material for: Effects of fasudil on glial cell activation induced by tooth movement
Source: Prog Orthod. 2024 Jul 22;25:33. doi: 10.1186/s40510-024-00518-2 (PMC11265063; doi:10.1186/s40510-024-00518-2)
Supplement: Supplementary file 1 — Supplementary Material 1 [file 40510_2024_518_MOESM1_ESM.pdf]

## Effects of fasudil on glial cell activation induced by tooth movement

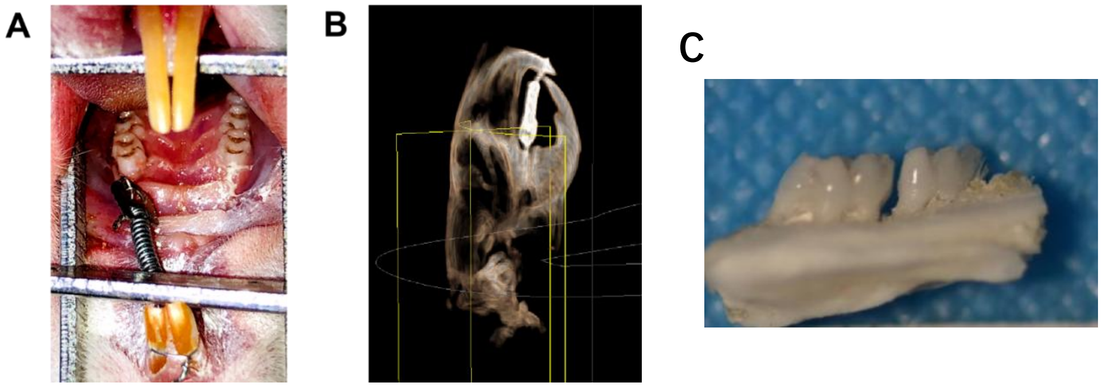


**Figure A1. Experimental tooth movement model in rats.**

(A-B) Experimental tooth movement model in rats (intraoral and lateral view of CBCT )

(C) Proximal mesial inclination of the first molar in rats.

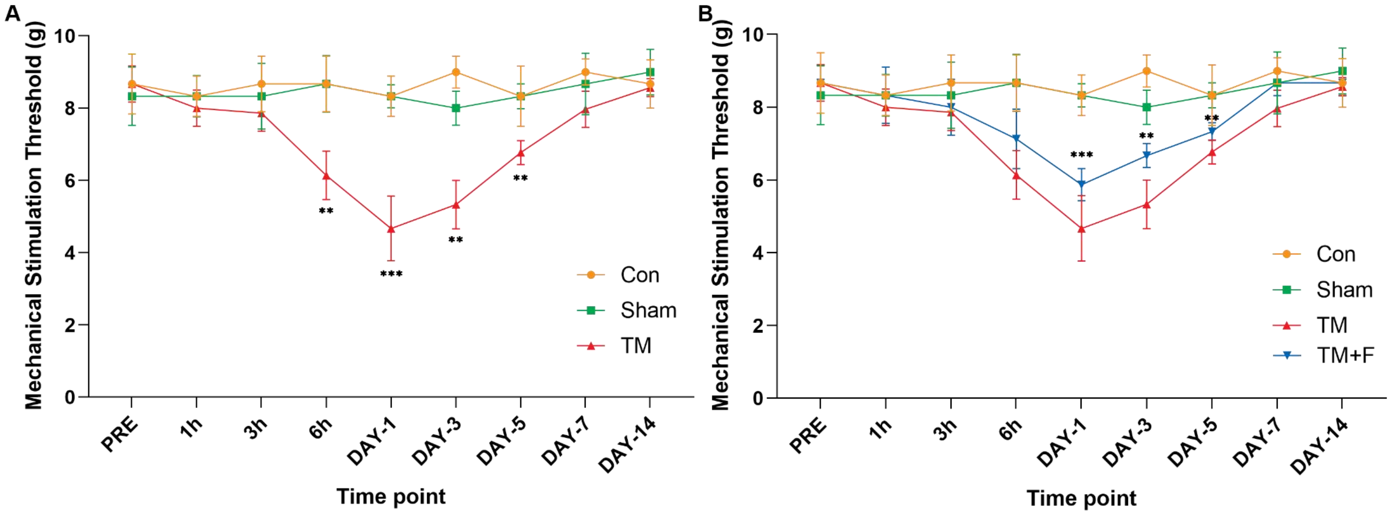


**Figure A2. The effect of experimental tooth movement on mechanical pain threshold in the orofacial region of rats.**

(A) Six hours after tooth movement, the mechanical pain threshold in the TM group was significantly lower than that in the Sham group, reaching its lowest point on the first day.

(B) In the fasudil-treated group (TM+F), the mechanical pain threshold increased, indicating the effectiveness of fasudil in alleviating tooth movement pain. All data are presented as the mean ± standard error (n = 5). *p < 0.5, **p < 0.01, ***p < 0.001.

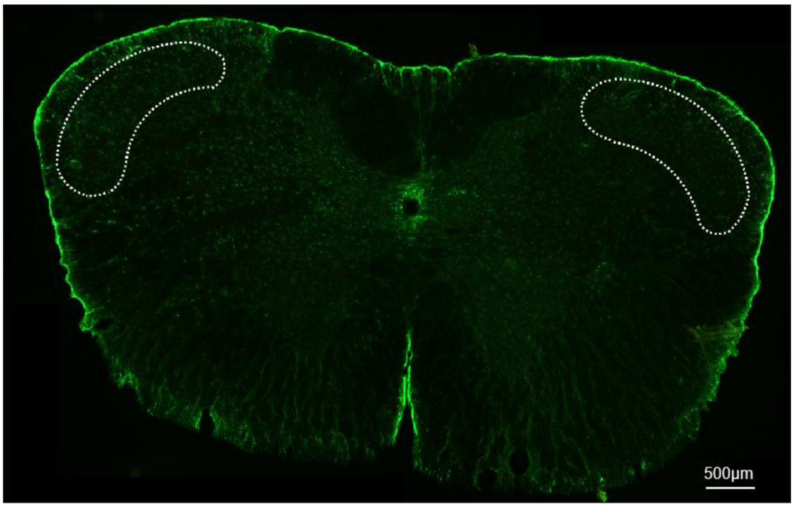


**Figure A3.** The dashed area represents the SPVC region in rats. All measurements and observations of immunofluorescence staining were performed in this region.

Table A1 Distance of Mesial Movement of the First Molar in TM and TM+F Groups

| GROUP | 7d | 14d |
|---|---|---|
| TM | 0.56±0.015 | 0.68±0.026 |
| TM＋F | 0.59±0.004 | 0.70±0.013 |
| P | NS | NS |

**Table A1: The distances of tooth movement for both the TM and TM+F groups**

The distances were of tooth movement measured at 7 and 14 days using a gap-measuring ruler. The difference was not statistically significant.
